# Systemic Correlates of Angiographic Coronary Artery Disease

**DOI:** 10.1371/journal.pone.0004322

**Published:** 2009-01-30

**Authors:** José Pedro L. Nunes, João Carlos Silva

**Affiliations:** 1 Faculdade de Medicina da Universidade do Porto, Porto, Portugal; 2 Hospital São João, Porto, Portugal; National Institute of Child Health and Human Development/National Institutes of Health, United States of America

## Abstract

Coronary angiography allows a direct evaluation of coronary anatomy. The aim of the present investigation was to search for correlations between the magnitude of coronary artery disease, as assessed by angiography, and a number of systemic parameters. A group of 116 patients (80 male, 36 female) with coronary heart disease diagnosed by angiography, aged 62.0±10.5 years, was the subject of an observational study. Correlation and linear regression analysis using coronary artery disease burden (CADB - sum of the percentage of the luminal stenosis encountered in all the lesions of the coronary arterial trees) as dependent variable, and age, sex, plasma calcium, phosphorus, magnesium, glucose, HDL cholesterol, LDL cholesterol, triglycerides, uric acid, estimated glomerular filtration rate and body mass index as independent variables, were carried out. Significant correlation values versus CADB were seen with age (r 0.19, p 0.04), uric acid (r 0.18, p 0.048) and fasting plasma glucose (r 0.33, p<0.001). Linear regression analysis, yielding a global significance level of 0.002, showed a significant value for glucose (p 0.018) and for sex (0.008). In conclusion, among several systemic parameters studied, plasma glucose was found to be correlated to coronary artery atherosclerosis lesions.

## Introduction

Coronary artery disease is a frequent and important disease, for which a number of risk factors have been identified. Coronary angiography allows a direct evaluation of coronary anatomy. The study of the magnitude of coronary artery disease in patients with confirmed disease may be of help in elucidating mechanisms underlying growth of coronary atherosclerosis lesions. This type of evidence may be of use, to be added to evidence on the risk of having the disease, notwithstanding the fact that some of the factors that increase risk may also increase growth.

Risk factors for ischemic heart disease include age, male sex, high plasma cholesterol, high plasma glucose and excessive weight [1]. More recently, a decrease in renal function was also noted to be associated to coronary artery disease [2].

An estimate of coronary artery disease burden (CADB) can be obtained by adding the degree of stenosis measured in every lesion found at angiography [2], and this was the method used in the present investigation. In a cohort previously studied, including 110 patients with acute coronary syndrome, relatively weak but significant correlations were seen between CADB and decreased renal function, on the one hand, and plasma calcium, on the other hand [2].

Atherosclerotic lesions frequently have not only lipid deposits, but also calcium deposits inside the arterial wall. There is evidence that a significant degree of similitude may exist between vascular calcification mechanisms and bone metabolism [3]–[5]. The relation between high plasma glucose and heart disease is also well established [6]. Diabetes mellitus, a state of chronic hyperglycemia, is frequently associated, not only to atherosclerosis, but also to other types of vascular calcification such as medial calcification, the same happening in patients with chronic renal failure [3]. Plasma glucose is known to be associated to the severity of coronary artery disease [7], [8]. Magnesium is an element that plays a role in multiple metabolic processes in physiology. Controversy exists, however, on the association between magnesium metabolism and cardiovascular disease, with different sets of data pointing in different directions [9]. Excessive weight/obesity is also currently seen as a risk factor for cardiovascular disease, and it could, at least in some cases, act as a cause for metabolic disturbances such as hyperglycemia [10]. Uric acid is another parameter that is currently receiving attention in the context of cardiac disease [11].

A number of biochemical parameters, including lipid fractions, may be changed in the context of acute coronary syndrome, and therefore it may be of interest to study outpatients, rather than inpatients with acute coronary syndrome, when performing a more comprehensive study including lipid fractions. The aim of the present investigation was, once again, to search for correlations between the magnitude of coronary artery disease, as assessed by angiography, and a number of systemic parameters: age, plasma calcium, phosphorus, magnesium, glucose, high density cholesterol (HDL), low density cholesterol (LDL), triglycerides, uric acid, estimated glomerular filtration rate and body mass index.

A possible interaction between lipid metabolism, calcium/phosphorus metabolism, magnesium, fasting plasma glucose, uric acid, renal dysfunction and excessive weight, on the one hand, and the magnitude of coronary artery lesions, and therefore mechanisms underlying lesion growth, on the other hand, was sought.

## Methods

A cohort of 116 patients (80 male, 36 female) with coronary heart disease diagnosed by angiography, aged 62.0±10.5 years, was the subject of an observational study. One hundred and fifteen patients were Caucasian and one was Asiatic. Patients were observed at an outpatient Cardiology clinic.

The study had an observational character, since biochemical studies were carried out after initial clinical evaluation (either before or after angiographic study), at a time when patients were under medical therapy that was not prescribed by any person involved in the present study. The research protocol (including the informed consent forms) was evaluated and approved by an independent ethics committee (Comissão de Ética do Centro de Saúde São João, Porto, Portugal). Patients were included if coronary artery disease was confirmed by angiography, performed after myocardial infarction or in the course of the study of angina pectoris. Exclusion criteria included serious systemic diseases, such as cancer and systemic infections.

Patients were evaluated under a protocol which included electrocardiogram, echocardiogram, and plasma measurement of total cholesterol, HDL (high density lipoprotein) cholesterol, triglycerides, creatinine, calcium, phosphorus, magnesium and uric acid (this latter parameter was not included in the initial analysis but was added at a later stage). LDL (low density lipoprotein) cholesterol values were calculated by the formula LDL = total cholesterol – HDL cholesterol – (0.2×triglycerides) [12]. Biochemical measurements (as well as the other tests) were performed under routine conditions, immediately after collection of blood and in a completely blind way in what concerns the present study. Additional diagnostic tests were performed according to each case. One biochemical set of data was taken for study in each patient, corresponding to the initial evaluation of the patient. Body mass index (BMI) was calculated by use of the formula weight (Kg)/height (m)^2^.

Quantitative evaluation of coronary arteriography was performed in all patients in two orthogonal views, either in the context of acute coronary syndrome or study of angina pectoris. The percent stenosis were calculated as the mean of the values obtained in the two views. Coronary artery disease burden (CADB) was estimated as the sum of the percentage of the luminal stenosis encountered in all the lesions of the coronary arterial trees, as previously reported [2].

Estimated Glomerular filtration rate (eGFR; milliliters per minute per 1.73 m^2^) was calculated according to the abbreviated Modification of Diet in Renal Disease (MDRD) study equation: GFR = 186×(serum creatinine)^−1.154^×(age)^−0.203^×0.742 (in women), as described in the corresponding K/DOQI Clinical Practice Guidelines [13]–[15].

As the patients were studied as outpatients, angiography and clinical/biochemical evaluation were not performed at the same moment in time. The average number of months between angiography and biochemical evaluation was 8.6±8.2, corresponding to the fact that a number of patients underwent coronary artery bypass surgery during that time interval.

Plasma samples were taken from patients either under lipid-lowering drug therapy (98 samples) or not under such therapy (18 samples). Statins (3-hydroxy-3-methyl-glutaryl coenzyme A reductase inhibitors) were used in 96 cases treated for dyslipidemia, in some cases in association to other types of drugs, whereas a fibrate was used as the sole lipid-lowering drug in two patients. Antidiabetic drugs were used in 28 patients, including insulin in nine patients and several types of oral drugs in the remaining cases. Data were missing for BMI in 3 patients.

Taking into consideration a possible interaction between glucose metabolic pathways and magnesium, possibly at the insulin receptor level, a composite parameter glucose/magnesium product was constructed by multiplying the values for each parameter.

### Statistical analysis

Data are presented as arithmetic mean and standard deviation. Correlations between the various parameters under study were calculated by using the Spearman correlation coefficient. Multiple linear regression, taking as dependent variable the estimation of CADB and as independent variables sex, age, plasma calcium, phosphorus, magnesium, glucose, HDL, LDL, triglycerides, uric acid, estimated glomerular filtration rate and body mass index, was performed, with the calculation of the overall probability and subsequent calculation of the individual probabilities for each independent variable. In order to perform linear regression study, all data was the subject of base 10 logarithmic transformation, except for sex (which was entered as either 1 for male and 2 for female patients). Pairs of means were compared by using the Mann- Whitney U test. Probability values<0.05 were considered significant. Statistical calculations were made by using the Statistical Package for the Social Sciences software (version 16.0).

## Results

Mean values for the different parameters under study are shown in Table 1. Correlation (univariable) analysis showed a number of significant correlations to exist. Significant correlation values versus CADB were seen with age (r 0.19, p 0.04), uric acid (r 0.18, p 0.048) and fasting plasma glucose (r 0.33, p<0.001).

**Table 1:** Descriptive statistics for a number of parameters measured in 116 patients with cardiovascular disease.

	Mean	STD	N
**Age**	62.0	10.5	116
**Ca**	2.37	0.10	116
**P**	33.2	5.1	116
**Mg**	1.64	1.31	116
**HDL**	48.2	11.6	116
**LDL**	123.6	41.8	116
**TG**	157.7	95.2	116
**Glucose**	118.9 (6.60)	50.1 (2.78)	116
**BMI**	27.8	4.9	113
**eGFR**	77.3	15.6	116
**Uric acid**	56.9	15.8	116

Age presented in years. Ca- plasma calcium, mmol/l; P- plasma phosphorus, mg/L; Mg- plasma magnesium, mEq/l; HDL- high density lipoprotein cholesterol, mg/dl; LDL- low density lipoprotein cholesterol, mg/dl; TG- triglycerides, mg/dl; Glucose- fasting plasma glucose, mg/dl (mmol/l); BMI- body mass index (Kg/m^2^); eGFR- estimated glomerular filtration rate, ml/min/1.73 m^2^; uric acid- plasma uric acid, mg/L; STD – Standard deviation; N- number.

Linear regression analysis (Table 2), yielding a global significance level of 0.002, showed a significant value for glucose (p 0.018) and for sex (0.008).

**Table 2:** Linear regression analysis, taking CADB (after base 10 logarithmic transformation) as dependent parameter and 10 parameters as independent parameters, as measured in 116 patients with coronary artery disease.

Dependent variable	CADB	
	Standardized Coefficients (Beta)	ANOVA p = 0.002 (F 2.876)
		p
**HDL**	−0.14	0.21
**LDL**	0.08	0.48
**TG**	0.09	0.35
**Ca**	0.06	0.51
**P**	0.05	0.58
**Mg**	0.09	0.34
**Gluc**	0.25	0.018
**Age**	0.06	0.54
**eGFR**	−0.16	0.14
**BMI**	−0.05	0.63
**Uric acid**	0.01	0.95
**Sex**	−0.31	0.008

HDL – HDL cholesterol; LDL – LDL cholesterol; TG – triglycerides; Ca – calcium; P – phosphorus; Mg- magnesium; Gluc- fasting glucose; eGFR- estimated glomerular filtration rate; BMI- body mass index; uric acid- plasma uric acid; p- probability.

The use of the composite parameter, glucose/magnesium product, also yielded a significant correlation with CADB (r 0.37, p<0.001).

Subgroup analysis, by dividing patients by sex (male/female) showed female patients to have lower mean values for CADB and uric acid, and higher mean values for BMI, HDL cholesterol, LDL cholesterol and plasma phosphorus (Table 3).

**Table 3:** Subgroup analysis (male/female) - mean±standard deviation.

Variable	Male (n = 80)	Female (n = 36)	P
**CADB**	314.4±169.4	206.4±144.7	0.001
**HDL**	46.4±10.6	52.3±12.8	0.014
**LDL**	116.9±35.8	138.5±50.1	0.034
**TG**	159.8±103.2	153.1±75.4	0.67
**Ca**	2.37±0.10	2.36±0.10	0.92
**P**	32.1+4.9	35.7+4.6	<0.001
**Mg**	1.65±0.13	1.61±0.13	0.21
**Glucose**	118.7±44.3	119.3±61.7	0.33
**Age**	62.5±10.2	60.8±11.2	0.49
**eGFR**	78.0±15.6	75.6±15.7	0.54
**BMI**	27.0±4.0	29.8±6.2	0.042
**Uric acid**	60.6±15.5	48.7+13.2	<0.001

Age presented in years. CADB- coronary artery disease burden; Ca- plasma calcium, mmol/l; P- plasma phosphorus, mg/L; Mg- plasma magnesium, mEq/l; HDL- high density lipoprotein cholesterol, mg/dl; LDL- low density lipoprotein cholesterol, mg/dl; TG- triglycerides, mg/dl; Glucose- fasting plasma glucose, mg/dl; BMI- body mass index (Kg/m^2^); eGFR- estimated glomerular filtration rate, ml/min/1.73 m^2^; uric acid- plasma uric acid, mg/L; N- number; p – probability (Mann- Whitney U test).

## Discussion

Coronary artery atherosclerotic lesions frequently involve the deposition of lipids and of calcium. Factors such as obesity, diabetes mellitus and renal dysfunction may also play a role in coronary atherosclerosis. In the present investigation, standard coronary angiography was used as a means to estimate coronary artery disease burden, by adding the values corresponding to each lesion found [2]. Age, uric acid and fasting plasma glucose were noted to be significantly correlated to CADB in univariate analysis. Linear regression (multivariate) analysis, however, only showed one of these parameters (fasting plasma glucose) to be associated to CADB, the same happening with sex.

All patients under study in the present investigation had coronary artery disease, and therefore no evaluation of the risk of acquiring the disease was carried out, although some of the parameters under study do act as risk factors. The present data may be of relevance in what concerns mechanisms favouring the growth of atherosclerosis lesions, since the parameter under evaluation (CADB) aimed at assessing the magnitude of the total burden of disease.

Lipids accumulate in arterial walls in atherosclerosis. In the present study, we could find no evidence of an association between lipid fractions and CADB. Most patients were treated with lipid-lowering drugs, and this may be one of the reasons behind these negative findings, particularly in what concerns LDL cholesterol. In the present study, HDL cholesterol levels were also not correlated to CADB, although previous studies have been shown HDL to be negatively associated with the importance of coronary artery disease, whereas no such relation was noted involving LDL cholesterol [16]–[17].

Calcium is yet another substance that is frequently found in atherosclerotic lesions. In a previous study carried out in patients with acute coronary syndrome [2], a relatively weak but significant correlation was noted between plasma calcium and CADB. In the present study, however, no significant correlation with CADB was found either with calcium or phosphorus. The mean plasma calcium measured in the present investigation (2.37 mmol/l, n = 116) was, in fact, significantly higher than in the previous study (2.20 mmol/l, n = 110), indicating that, as previously reported [18], plasma calcium values measured in inpatients may be different from values measured in outpatients, and may not hold precisely the same physiological significance.

Renal dysfunction could act as a risk factor for coronary artery disease [2]. However, it is unclear if renal dysfunction acts as a cause for accelerated coronary artery disease or if it merely acts as a surrogate marker for the overall systemic vascular system status [2]. Obesity also acts as a risk factor for cardiovascular disease. Previous studies have shown that increased BMI values may be associated to lower coronary artery disease burden, a finding that may depend on the fact that such patients are frequently studied at an earlier age [19]. In the present investigation, no relation was noted between BMI and CADB, neither in univariate nor in linear regression analysis.

Diabetes mellitus is a well known risk factor for coronary artery disease [6], as well as cardiovascular disease in general [20]. Glucose metabolism has been shown to correlate with the angiographic importance of coronary artery disease, data in good agreement with the present results [7]–[8]. Diabetes mellitus has also been shown to be associated to coronary artery calcification in asymptomatic subjects [21]. Elevated fasting plasma glucose in type II diabetes mellitus is known to be frequently associated to elevated plasma levels of plasma insulin. In the present results, plasma glucose showed a significant relation versus CADB in both univariate and multivariate analysis, indicating that glucose metabolic pathways may play a role in the growth of atherosclerotic lesions. This may happen not necessarily through the action of elevated glucose levels, but perhaps through the action of insulin. Insulin could act not only on insulin receptors but also on insulin/IGF receptor hybrids [22], and IGF receptors could play a role in cell proliferation [22].

Magnesium seems to act as a cofactor for insulin receptor-associated tyrosine kinase activity [22], one among a large number of physiological actions of this element. Considerable controversy exists concerning the role of magnesium in cardiovascular disease [9]. Uric acid has been suggested to play a role in cardiorenal disease, namely in arterial hypertension, but also in coronary artery disease [11]. In the present investigation, plasma uric acid was correlated to CADB in univariate but not in linear regression analysis.

In the context of the present investigation, one may speculate that higher plasma glucose, probably in the presence of elevated plasma insulin, could be associated to a growth-stimulating effect on atherosclerotic lesions, perhaps involving magnesium as a cofactor for insulin-stimulated growth. This latter phenomenon, at least in vascular smooth muscle cells, may depend on the stimulation of intracellular pathways involving mitogen-activated protein kinases [23] (for review please see ref. 22). Recent data show that the use of an insulin sensitizer (pioglitazone) was associated with a lower rate of progression of coronary atherosclerosis than the use of an insulin secretagogue (glimepiride) in patients with type 2 diabetes [24], data that may be interpreted taking into consideration the line of reasoning presented above.

The data concerning the subgroup analysis (male/female) must be viewed with great caution, since it is not clear that the clinical background is similar for both subgroups of patients. The well-known finding of higher HDL cholesterol in female patients [25] was seen, but it is unclear if the remaining findings (females with higher BMI, LDL cholesterol, and phosphorus, lower uric acid and CADB) could represent a common pattern in patients with coronary atherosclerosis.

Limitations of the present study include the relatively small sample of patients. Angiography is a technique that detects major coronary arterial lesions, leaving important segments of diseased vessels unrecognized as such [26]. Patients were observed as outpatients at a cardiology clinic, and so several types of selection bias may be at play. The authors would also like to stress the limitations of not including treatment factors in the analysis as well as the variable time between biomarker and angiography measurements in the study population. Patients were frequently under the influence of drugs, both lipid-lowering and anti-diabetic. Thus, particularly in what concerns the LDL data, these results must be viewed with great caution. Several parameters were under study, and so a cautionary note on the findings in the background of tests on multiple parameters is warranted. Given these limitations, the present findings should be, preferably, confirmed by other studies.

In conclusion, among several systemic parameters studied, plasma glucose was found to be correlated to coronary artery atherosclerosis lesions.
